# Projected prevalence and incidence of dementia accounting for secular trends and birth cohort effects: a population-based microsimulation study

**DOI:** 10.1007/s10654-022-00878-1

**Published:** 2022-06-22

**Authors:** Chiara Celine Brück, Frank J. Wolters, M. Arfan Ikram, Inge M.C.M. de Kok

**Affiliations:** 1 Department of Public Health, Erasmus MC University Medical Center, Rotterdam, the Netherlands; 2 Department of Epidemiology, Erasmus MC University Medical Center, Rotterdam, the Netherlands

**Keywords:** Dementia, Alzheimer Disease, Incidence, Prevalence, Forecasting, Aging

## Abstract

There is need for accurate projections of the future dementia burden to prepare healthcare systems and policymakers. Existing projections only account for population ageing, not for observed declines in age-specific dementia incidence of 13% per decade. We developed a dementia microsimulation model that synthesizes population-based data from the Rotterdam Study with changes in demographics between birth cohorts from the early 1900s onwards. We determined dementia prevalence and incidence until 2050 for three different dementia incidence trend scenarios: (1) stable age-specific incidence, (2) linear decline by 13% per decade, (3) nonlinear declines averaging 13% per decade. Assuming a stable age-specific incidence resulted in a 130% increase in incidence and 118% in prevalence between 2020 and 2050. By contrast, the linearly declining trend resulted in substantially smaller increases of 58% in incidence (95%CI: 29–87%), and 43% in prevalence (95%CI: 13–66%), corresponding to 39% lower incidence and 36% lower prevalence by 2050 than in the stable-incidence scenario. Results for various non-linear declines fell between the stable and linear trend. The future burden of dementia is highly susceptible to achievable changes in age-specific incidence. Extension of previously established secular trends globally would reduce widely upheld projections of new dementia cases until 2050 by 39%.

**Supplementary information:**

The online version contains supplementary material available at 10.1007/s10654-022-00878-1.

## Introduction

Worldwide, more than 50 million people are currently living with dementia [1], which presents a significant public health issue [2] and substantial burden on health-care systems and society. Dementia is the seventh leading cause of death globally [3] and causes 30 million disability-adjusted life years (DALYs) annually [4]. Dementia incidence increases exponentially with age [5], and the overall incidence is therefore expected to increase due to demographic ageing of populations worldwide. However, several studies have now shown that the age-specific incidence of dementia has declined over the past decades, by about 13% per decade since 1990 [6, 7]. Although the underlying causes remain undetermined, improvements in for example management of cardiovascular disease and educational attainment are assumed to play a key role [8, 9]. As a result of the steep increase in incidence with age, relatively small delays in the onset of dementia may have substantial effects on incidence and prevalence [5, 10]. As such, the reported declines in age-specific incidence could have a large impact on the future burden of disease, but the effect of secular trends on absolute disease burden is undetermined.

In order to prepare healthcare systems and policymakers, the WHO estimated that population ageing will cause global dementia prevalence to triple in the coming 30 years, from 47 million in 2015 to 150 million in 2050 [1]. Similarly, several modeling studies have investigated the effect of population ageing on global and country-specific prevalence and concluded that dementia prevalence will double every 20 years [11–13]. However, these projections do not account for secular trends in dementia incidence [8, 14], due in part to the lack of available models that account for secular trends in both demographic build, life-expectancy, and dementia incidence in populations. Microsimulation models are particularly equipped to allow for the inclusion of trends in dementia risk in addition to demographic ageing, due to their individual-level model structure [15]. As such, microsimulation models can provide valuable projections of the future burden of dementia to inform policy priorities and resource allocation.

We therefore developed a microsimulation model, with the aim of determining the change in the prevalence and incidence of dementia until 2050, accounting for long-term secular trends and birth cohort effects.

## Methods

For this study, we used population-based data from the Rotterdam Study [16] and the governmental national institute for demographics ‘Statistics Netherlands’ [17] to develop a dementia microsimulation model, based on the validated Microsimulation Screening Analysis (MISCAN) model from cancer research [18].

## Data

Age- and sex-specific dementia and mortality data were derived from participants of the Rotterdam Study, an ongoing population-based cohort study of 14,926 individuals aged > 40 years who reside in the Ommoord suburb of Rotterdam, the Netherlands. Details of the study design and of case ascertainment for dementia and mortality have been described previously [10, 16]. For the current study, we included all 10,209 participants who were alive and non-demented before start of the fourth examination cycle on 1 January 2002. Information on vital status was obtained regularly from the municipal health authorities in Rotterdam. Participants were screened for dementia at each four-yearly center visit using the Mini-Mental State Examination and the Geriatric Mental Schedule organic level, and screen-positives underwent further investigation and informant interviews, including the Cambridge Examination for Mental Disorders of the Elderly. All participants also underwent routine cognitive assessment, including a verbal fluency test (animal categories), 15-word learning test, letter-digit substitution task, Stroop test, and Purdue pegboard task. In addition, they were under continuous surveillance for the detection of interval cases between center visits, through electronic linkage of the study base with medical records from general practitioners and the regional institute for outpatient mental healthcare. A consensus panel headed by a consultant neurologist established the final diagnosis in accordance with standard criteria for dementia (DSM-III-R). As such, 1118 of 10,209 participants were diagnosed with dementia during follow-up until 1 January 2016.

Statistics Netherlands is recognized as a leading National Statistical Institute and is regularly peer reviewed by Eurostat [19]. The accuracy and completeness of its data are continuously verified by the Advisory Council of the Dutch Director General [20]. We constructed cohort specific birth and life tables for 10-year birth cohorts between 1910 and 1989. The birth tables were based on age- and sex-specific death rates [21] and population estimates [22] to represent the Dutch population. The life tables were based on historic and predicted age- and sex-specific death rates for the years 1910 to 2089 [23, 24] and adjusted for dementia-related mortality [25] (Appendix A). The distribution of individuals across the birth cohorts is based on the ratio between the historic births of the given cohort and the births in all cohorts [24]. The gender distribution in the birth cohorts is based on the historic gender distribution in the given decade [17]. Improvements in survival of birth cohorts in the Netherlands are similar to those observed in other high-income countries [26], including the United States and the United Kingdom (see Appendix A for a direct comparison).

## Statistical model

MISCAN-Dementia, coded in Python 3.9, is a stochastic, semi-Markov microsimulation model that predicts how dementia incidence and prevalence will develop until 2050. The model simulates the life histories of 10 million individuals, representative of the Dutch population in 10-year birth cohorts from 1910 to 1989. The model allows for unidirectional stage transitions between cognitively normal, mild cognitive impairment (MCI), dementia, and death due to dementia. MISCAN-Dementia is a time-to-event model; hence transitions are determined by the duration of the stages. In any stage, an individual can also die of other causes. Differential mortality of individuals with dementia is implicitly introduced by the duration of the dementia stage, which is often shorter than the remaining life expectancy without dementia. The model was calibrated to fit the age-specific dementia incidence data of the Rotterdam Study. Model fit was excellent (Figure C1 in Appendix C). Further details on the model development and specification can be found in Appendix B.

## Trend analysis

### Base case analysis

As base case scenarios, we calibrated a stable and a linear trend in age-specific dementia incidence to fit the observed incidence data from the Rotterdam Study [16]. First, we determined prevalence and incidence for the scenario in which age-specific incidence remains stable (‘no decline’ trend in Table 1). Every birth cohort was calibrated to fit the age-specific dementia incidence rates of the Rotterdam Study. Second, we calibrated the same model using a linear decline in the age-specific incidence of 13% per decade, as was recently observed in a meta-analysis of North-American and European cohorts (‘linear trend’ in Table 1) [6]. Since we used Rotterdam Study data from 2002 onwards, the 1940s birth cohort was the most suitable ‘base cohort’ for participants aged 60–69 as most participants of that age group in the data collection period were born during the 1940s. Similarly, the 1930s birth cohort was most suitable for those aged 70–79, 1920s for individuals of 80–89 years, and 1910s for participants of 90 years and older. Base cohorts have incidence rates equal to the age group estimate from the Rotterdam Study. Compared to the base cohort, later birth cohorts are calibrated with a 13% lower age-specific dementia incidence, whereas earlier birth cohorts are assumed to have a 13% higher incidence. For the different trend scenarios, dementia incidence rates and prevalence were calculated for the calendar years 1995 to 2050, using (projected) demographic data of the Dutch population > 65 years for the same period [22, 27].

**Table 1:** Trend scenarios expressed in percentage change between birth cohorts

Trend Type	No.	1910-19 to 1920-29	1920-29 to 1930-39	1930-39 to 1940-49	1940-49 to 1950-59	1950-59 to 1960-69	1960-69 to 1970-79	1970-79 to 1980-89
**No decline**	**1.0**	0%	0%	0%	0%	0%	0%	0%
**Linear**	**2.0**	-13%	-13%	-13%	-13%	-13%	-13%	-13%
**Linear (low)**	**2.1**	-7%	-7%	-7%	-7%	-7%	-7%	-7%
**Linear (high)**	**2.2**	-19%	-19%	-19%	-19%	-19%	-19%	-19%
**Nonlinear stable**	**3.0**	-13%	-13%	-13%	-10%	-7%	-5%	-4%
**Nonlinear stable**	**3.1**	-13%	-13%	-13%	0%	0%	0%	0%
**Nonlinear decelerating**	**4.0**	-18%	-13%	-8%	-5%	-3%	-1%	0%
**Nonlinear decelerating**	**4.1**	-18%	-13%	-8%	0%	0%	0%	0%
**Nonlinear decelerating**	**4.2**	-22%	-11%	-5%	-4%	-3%	-2%	-1%
**Nonlinear decelerating**	**4.3**	-22%	-11%	-5%	0%	0%	0%	0%
**Nonlinear decelerating**	**4.4**	-24%	-12%	-2%	0%	0%	0%	0%
**Nonlinear accelerating**	**5.0**	-8	-13%	-18%	-13%	-8%	-5%	-3%
**Nonlinear accelerating**	**5.1**	-5	-11%	-22%	-11%	-5%	-3%	-1%
**Nonlinear accelerating**	**5.2**	-2	-12%	-24%	-12%	-2%	0%	0%

### Sensitivity analysis

We performed two types of sensitivity analyses. First, we investigated the effect of varying the magnitude of the linear trend by repeating the analysis with the 95% confidence intervals (7–19%) of the linear trend estimate in the prior meta-analysis (‘linear low’ and ‘linear high’ in Table 1) [6]. Second, we relaxed the linearity assumption for the trend in incidence, examining three types of nonlinear trends: (1) linear decline across the 1910 to 1940 birth cohorts and decelerating or no decline across the later birth cohorts (‘nonlinear stable’ in Table 1); (2) decelerating decline across all birth cohorts (‘nonlinear decelerating’ in Table 1) and (3) accelerating decline across the 1910 to 1940 birth cohorts and decelerating decline across the later birth cohorts (‘nonlinear accelerating’ in Table 1). The nonlinear trends were chosen such that the overall change in incidence rate between the 1910 and 1940 birth cohorts and the average incidence rate of the 1910 and 1940 birth cohorts were similar to the overall change and average incidence rate of the linear trend for the same birth cohorts.

## Results

### Base case analysis

**Fig. 1:**
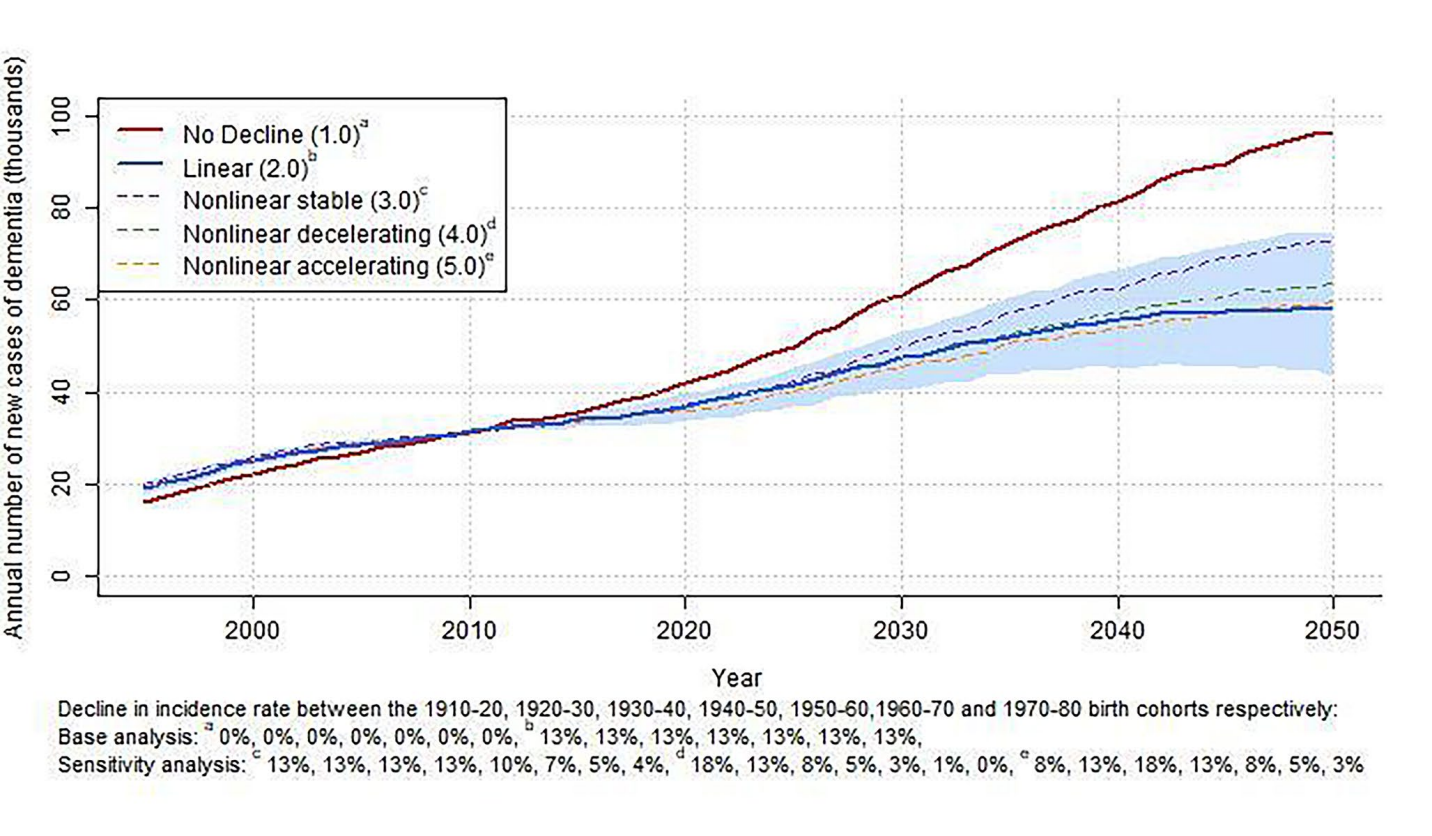
Total dementia incidence in the Netherlands per calendar year assuming no trend, a linear trend of 13% (shaded area 95% CI) and three nonlinear trends

**Fig. 2:**
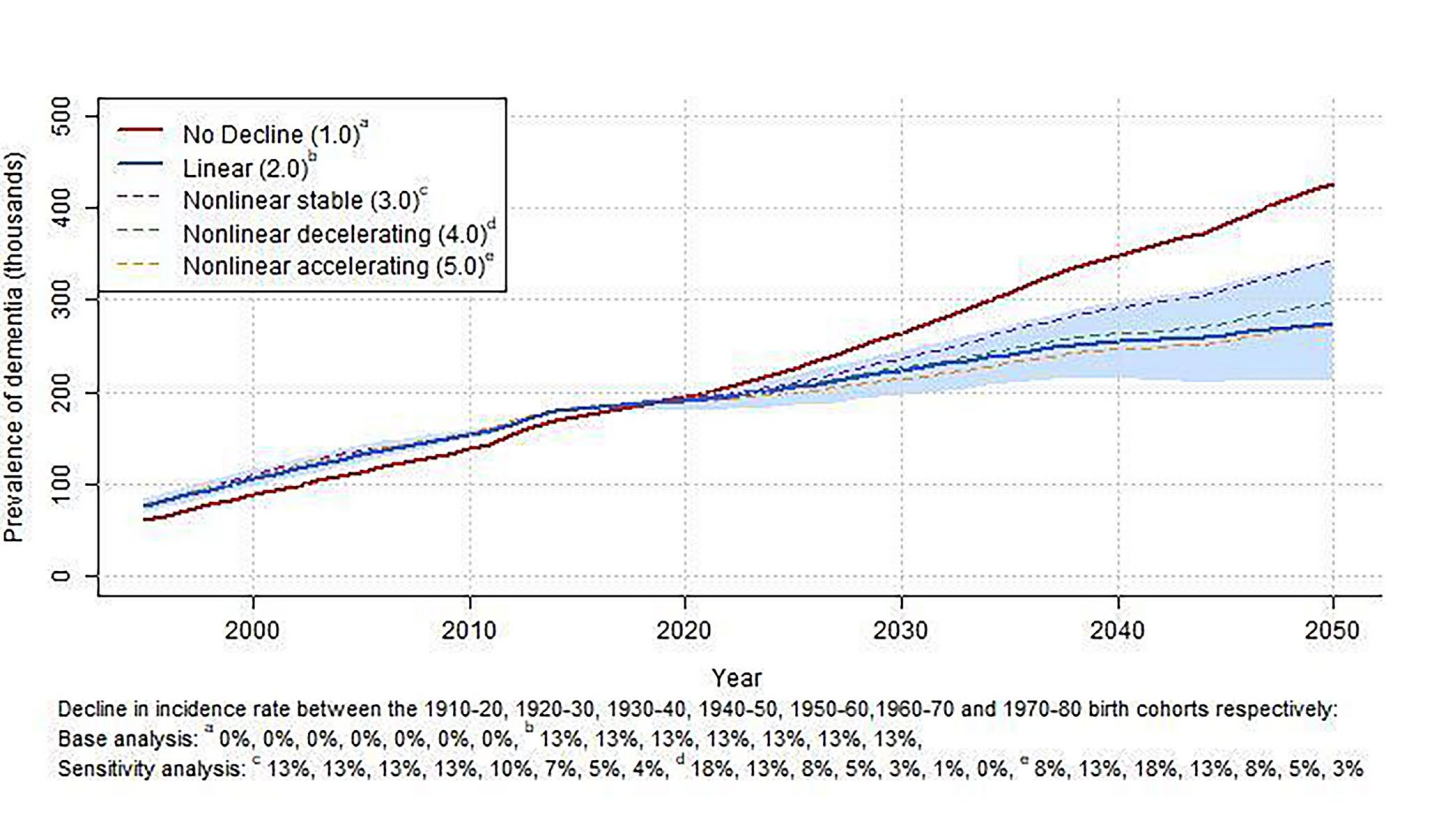
Total dementia prevalence in the Netherlands per calendar year assuming no trend, a linear trend of 13% (shaded area 95% CI) and three nonlinear trends

Figures 1 and 2 show the total incidence and total prevalence of dementia in the Dutch population per calendar year for the various assumed trends. Model calibration was good, as can be appreciated from the similar number of incidence cases in the different trend scenarios in the period 2002 to 2016 (i.e., the last year of calibration). The lines from 2017 to 2050 show the projected effect of the various secular trends on incidence and prevalence from 2016 onwards. Assuming a continuing, linearly declining trend resulted in significantly lower incidence and prevalence projections than assuming no decline (Figs. 1 and 2). This difference increased with calendar time, such that by 2030, the linear trend simulation projected 22% fewer incident cases and 15% fewer prevalent cases of dementia than the projections assuming no decline. By 2050, this difference amounted to 39% for the incidence and 36% for the prevalence (Table 2).

**Table 2:** Incidence and prevalence projections for 2030 and 2050 (per 1000 person years above age 65)

				2030						2050			
	**Trend Scenario**	**No.**		**Rate**	**Δ no trend**	**Δ linear**	**Increase 2020 to 2030**		**Rate**		**Δ no trend**	**Δ linear**	**Increase 2020 to 2050**
**Incidence**	No trend	1.0		14.4	*ref*	28%	**45%**		19.9		*ref*	65%	**130%**
	Linear	2.0		11.2	-22%	*ref*	**29%**		12.1		-39%	*ref*	**58%**
	Nonlinear (stable)	3.0		11.3	-22%	0%	**28%**		13.2		-34%	9%	**71%**
	Nonlinear (dec.)	4.0		11.8	-18%	5%	**34%**		15.0		-25%	24%	**96%**
	Nonlinear (acc.)	5.0		10.8	-25%	-4%	**26%**		12.3		-38%	2%	**65%**
**Prevalence**	No trend	1.0		62.5	*ref*	18%	**35%**		88.2		*ref*	56%	**118%**
	Linear	2.0		52.9	-15%	*ref*	**17%**		56.5		-36%	*ref*	**43%**
	Nonlinear (stable)	3.0		53.5	-14%	1%	**18%**		61.4		-30%	9%	**55%**
	Nonlinear (dec.)	4.0		55.7	-11%	5%	**22%**		70.8		-20%	25%	**77%**
	Nonlinear (acc.)	5.0		50.7	-19%	-4%	**13%**		56.5		-36%	0%	**44%**

The projected scenario with no decline resulted in a more than doubling of dementia incidence and prevalence until 2050 (+ 130% incident cases, and + 118% prevalent cases). According to the linear declining trend, dementia burden still increased from 2020 to 2050, but by a much smaller magnitude (+ 58% incident cases, and + 43% prevalent cases).

### Sensitivity analysis

The blue shaded area in Figs. 1 and 2 represents the variation in projected dementia burden within the 95% confidence limits of the linear trend. Assuming the lower bound of the 95% confidence interval of the linear trend estimate results in higher incidence and prevalence projections compared to the linear trend (+ 28% and + 25% in 2050, Table C2 and C3 in Appendix C), but these were still substantially lower than the no decline projections (Figs. 1 and 2). Assuming the upper bound resulted in 26% lower incidence and 22% lower prevalence projections by 2050, compared to the linear trend.

Results of the main sensitivity analyses with stable (3.0), decelerating (4.0), and accelerating (5.0) trends all fell within the 95% confidence limits of the linear trend (Figs. 1 and 2). The stable (3.0) and decelerating (4.0) nonlinear trends thereby resulted in higher incidence and prevalence projections than the linear trend (+ 9 to + 25% in 2050, Table 2) but well below the no decline projections (-20 to -34% in 2050). The accelerating (5.0) nonlinear trend resulted in roughly equal incidence and prevalence projections compared to the linear trend (-4% in 2030, 0% to + 2% in 2050). Comparisons of other nonlinear trends from Table 1 with the linear trends scenario were broadly similar (Appendix C Figures C2 and C3, Tables C2 and C3), with one important exception: the nonlinear scenarios in which decline in incidence decelerated to 0 from 1940 onwards (i.e., scenarios 3.1, 4.1, 4.3, and 4.4), projected incidence and prevalence by 2050 were closer to the no decline trends than to the linear trend scenario from the main analysis (Tables C2 and C3).

## Discussion

Using long-term population-based data from the Netherlands, we showed that incorporation of previously established dementia incidence trends in a microsimulation study has major effects on the projected dementia burden until 2050. The overall disease burden is set to increase due to aging of the population, but accounting for changes in life-expectancy and population structure, a continued 13% decline in age-specific dementia incidence per decade would result in 36% lower prevalence than widely upheld projections. These projections foster hope for curbing the dementia epidemic in high-income countries, and underline the need to similarly fulfil preventive potential worldwide.

Several studies, including the widely cited World Alzheimer reports, have reported on the basis of an ageing population, that the number of people living with dementia will triple between 2015 and 2050 [1, 13]. These projections, not accounting for secular trends in dementia incidence, are in line with the projection in the ‘no decline’ scenario in the current study. Incorporation of established temporal trends in incidence rates, however, substantially attenuates the projected increase in the burden of dementia. Relatively small changes over time in dementia incidence can have a major impact on the burden of disease at a population level, with important implications for care organization. Findings from this microsimulation model can inform healthcare providers and policymakers on preparing the healthcare system for the coming years.

It is important to note that projections are based on observed incidence trends in high income countries only. While this is highly relevant in light of anticipated increases in dementia prevalence in for example the United States [11, 12, 28], Canada [29], the United Kingdom [30], and Spain [31], the global increase in the prevalence of dementia will affect predominantly those in low- and middle-income countries, as the elderly population will grow faster in low- and middle-income countries than in high-income countries [32]. Moreover, consistent declines in dementia incidence in high-income countries do not necessarily apply to other regions. In fact, preliminary evidence from Asia and Africa shows stable or even increasing dementia incidence rates [33–35]. Additional high-quality data on incidence trends across the globe are needed to inform models for more precise projections in currently underrepresented regions.

The projections from our model encourage redoubled efforts to sustain the declining incidence trends into the 21st century. This is especially important given the less optimistic results from sensitivity analyses, in which declines in dementia incidence were not sustained for those born after 1950– a cohort for which only limited population-based dementia incidence data is yet available. The precise causes of the declining trends in dementia incidence rates have not been established, but likely include a combination of improvements in cardiovascular health [36, 37], educational attainment [38, 39], and lifestyle [40, 41], and maternal and child health. Prevention initiatives are needed to promote brain health worldwide, if we are to achieve the full potential of dementia prevention. Given the lag time between improvements (or deteriorations) in exposure and dementia onset, it may take years or even decades before public health improvements in prenatal development or reduction of midlife hypertension translate into changes in dementia incidence. As noted before and corroborated by present projections, declining trends may be easily overturned by increases in, for example, the global prevalence of obesity and hypertension [42]. It is therefore important to gain further insight into the causes and development of the declining trends, to be able to make better predictions of the future incidence and prevalence of dementia, as well as to determine the focus of prevention initiatives.

To our knowledge, MISCAN-Dementia is the first microsimulation model that provides projections for the general population and evaluates changes in dementia risk across birth cohorts. MISCAN-Dementia synthesizes all available information on the natural history of dementia by using input values taken from observed population-based data and peer-reviewed literature. Although we believe the model provides valid scenarios for the future dementia burden, some limitations should be taken into account. First, this model was developed on the basis of Dutch data. Although changes in population structure and relative reductions in dementia incidence are similar across high-income countries [6, 26], this may hamper generalizability of the projections. Also, the Rotterdam Study population is predominantly White. Future calibrations of the MISCAN-Dementia model using data from the U.S. and other geographical regions may further refine global projections. Second, any model is a simplification of reality, the accuracy of which depends on the underlying structure and assumptions about the demographics of the simulated birth cohorts and the (heterogeneous) natural history of dementia. Although differences in demographics between birth cohorts were explicitly modeled, factors such as migration patterns or the effect of pandemics (i.e. SARS-CoV-2) were not taken into account. In order to increase validity, we used population-representative data, and created insight in the uncertainty by various sensitivity analyses. The prevalence projections of this study (~ 190,000 cases in 2019) compare well to existing estimates in the Netherlands based on GP registries (~ 184,000 in 2019) [43] and healthcare use claims data (~ 250,000 in 2017) [44]. Nevertheless, repeated updates of the model will be needed as additional disease course and incidence trend data become available (i.e. from ongoing cohort studies).

To conclude, changes in dementia incidence between birth cohorts need to be taken into account to provide realistic projections of the future burden of dementia. We developed a microsimulation model that is capable of projecting secular trends on future dementia incidence and prevalence. Applied to the Dutch population, this results in projections of incidence and prevalence that are 20–39% lower than currently assumed. These projections allow policymakers, healthcare providers and researchers to ensure appropriate allocation of funds, training of healthcare personnel and accurately estimating potential effects of future interventions, and emphasize the need for efforts to fulfill the potential for dementia prevention globally.

## Electronic supplementary material

Below is the link to the electronic supplementary material.

Supplementary Material 1

## Data Availability

Not applicable.

## References

[1] Patterson C World Alzheimer Report 2018. The state of the art of dementia research: New frontiers2018 June 30th, 2021.

[2] World Health Organization. Global action plan on the public health response to dementia 2017–20252017.

[3] World Health Organization. The top 10 causes of death. 2020. https://www.who.int/news-room/fact-sheets/detail/the-top-10-causes-of-death. Accessed June 17th, 2021.

[4] Nichols E, Szoeke CEI, Vollset SE (2019). Global, regional, and national burden of Alzheimer’s disease and other dementias, 1990–2016: a systematic analysis for the Global Burden of Disease Study 2016. Lancet Neurol.

[5] Licher S, Darweesh SKL, Wolters FJ (2019). Lifetime risk of common neurological diseases in the elderly population. J Neurol Neurosurg Psychiatry.

[6] Wolters FJ, Chibnik LB, Waziry R (2020). Twenty-seven-year time trends in dementia incidence in Europe and the United States: The Alzheimer Cohorts Consortium. Neurology.

[7] Satizabal CL, Beiser AS, Chouraki V, Chêne G, Dufouil C, Seshadri S (2016). Incidence of dementia over three decades in the Framingham Heart Study. N Engl J Med.

[8] Schrijvers EM, Verhaaren BF, Koudstaal PJ, Hofman A, Ikram MA, Breteler MM (2012). Is dementia incidence declining?: Trends in dementia incidence since 1990 in the Rotterdam Study. Neurology.

[9] Langa KM (2015). Is the risk of Alzheimer’s disease and dementia declining?. Alzheimers Res Ther.

[10] Wolters FJ, Tinga LM, Dhana K (2019). Life expectancy with and without dementia: a population-based study of dementia burden and preventive potential. Am J Epidemiol.

[11] Hebert LE, Weuve J, Scherr PA, Evans DA (2013). Alzheimer disease in the United States (2010–2050) estimated using the 2010 census. Neurology.

[12] Matthews KA, Xu W, Gaglioti AH (2019). Racial and ethnic estimates of Alzheimer’s disease and related dementias in the United States (2015–2060) in adults aged ≥ 65 years. Alzheimers Dement.

[13] Prince M, Bryce R, Albanese E, Wimo A, Ribeiro W, Ferri CP (2013). The global prevalence of dementia: a systematic review and metaanalysis. Alzheimers Dement.

[14] Wu YT, Beiser AS, Breteler MMB (2017). The changing prevalence and incidence of dementia over time - current evidence. Nat Rev Neurol.

[15] Norton S, Matthews FE, Brayne C (2013). A commentary on studies presenting projections of the future prevalence of dementia. BMC Public Health.

[16] Ikram MA, Brusselle G, Ghanbari M (2020). Objectives, design and main findings until 2020 from the Rotterdam Study. Eur J Epidemiol.

[17] Statistics Netherlands. Bevolking, huishoudens en bevolkingsontwikkeling; vanaf 1899. 2020. https://opendata.cbs.nl/statline/#/CBS/nl/dataset/37556/table?ts=1607289563297. Accessed December 6th, 2020.

[18] Habbema J, Van Oortmarssen G, Lubbe JTN, Van der Maas P (1985). The MISCAN simulation program for the evaluation of screening for disease. Comput Methods Programs Biomed.

[19] Gerry O’Hanlon. KS, Tomaz Smrekar. Peer review report on compliance with the code of practice and the coordination role of the national statistical institute - Netherlands2015.

[20] Statistics Netherlands. The Advisory Council. 2017. https://www.cbs.nl/en-gb/over-ons/organisation/the-advisory-council. Accessed June 17, 2021.

[21] Statistics Netherlands. Levensverwachting; geslacht, leeftijd (per jaar en periode van vijf jaren). 2020. https://opendata.cbs.nl/statline/#/CBS/nl/dataset/37360ned/table?ts=1600695663107. Accessed December 4th, 2020.

[22] Statistics Netherlands. Bevolking; geslacht, leeftijd en burgerlijke staat, 1 januari. 2020. https://opendata.cbs.nl/statline/#/CBS/nl/dataset/7461bev/table?ts=1608026559329. Accessed December 4th, 2020.

[23] Statistics Netherlands. Prognose periode-levensverwachting; geslacht en leeftijd, 2020–2070. 2020. https://opendata.cbs.nl/#/CBS/nl/dataset/84883NED/table?searchKeywords=leeftijd. Accessed March 18th, 2021.

[24] Human Mortality Database. University of California BU, and Max Planck Institute for Demographic Research (Germany). Netherlands. 2020. www.mortality.org. Accessed March 18th, 2021.

[25] Statistics Netherlands. Overledenen; doodsoorzaak (uitgebreide lijst), leeftijd, geslacht. 2020. https://opendata.cbs.nl/statline/#/CBS/nl/dataset/7233/table?ts=1615054270240. Accessed April 1st, 2021.

[26] Beltrán-Sánchez H, Subramanian S (2019). Period and cohort-specific trends in life expectancy at different ages: Analysis of survival in high-income countries. SSM-population health.

[27] Statistics Netherlands. Prognose bevolking; kerncijfers, 2020–2070. 2020. https://opendata.cbs.nl/statline/#/CBS/nl/dataset/84871NED/table?ts=1608116990760 Accessed December 16th, 2020.

[28] Brookmeyer R, Abdalla N, Kawas CH, Corrada MM (2018). Forecasting the prevalence of preclinical and clinical Alzheimer’s disease in the United States. Alzheimers Dement.

[29] Manuel DG, Garner R, Finès P (2016). Alzheimer’s and other dementias in Canada, 2011 to 2031: a microsimulation Population Health Modeling (POHEM) study of projected prevalence, health burden, health services, and caregiving use. Popul Health Metr.

[30] Wittenberg R, Hu B, Barraza-Araiza L, Rehill A. Projections of older people with dementia and costs of dementia care in the United Kingdom, 2019–20402019.

[31] Soto-Gordoa M, Arrospide A, Moreno-Izco F, Martínez-Lage P, Castilla I, Mar J (2015). Projecting Burden of Dementia in Spain, 2010–2050: Impact of Modifying Risk Factors. J Alzheimers Dis.

[32] Prince MJ (2015). World Alzheimer Report 2015: the global impact of dementia: an analysis of prevalence.

[33] Ohara T, Hata J, Yoshida D (2017). Trends in dementia prevalence, incidence, and survival rate in a Japanese community. Neurology.

[34] Li S, Yan F, Li G (2007). Is the dementia rate increasing in Beijing? Prevalence and incidence of dementia 10 years later in an urban elderly population. Acta Psychiatr Scand.

[35] Gao S, Ogunniyi A, Hall KS (2016). Dementia incidence declined in African-Americans but not in Yoruba. Alzheimers Dement.

[36] Wolters FJ, Zonneveld HI, Hofman A (2017). Cerebral perfusion and the risk of dementia: a population-based study. Circulation.

[37] Wolters FJ, de Bruijn RF, Hofman A, Koudstaal PJ, Ikram MA (2016). Cerebral vasoreactivity, apolipoprotein E, and the risk of dementia: a population-based study. Arterioscler Thromb Vasc Biol.

[38] Ngandu T, von Strauss E, Helkala E-L (2007). Education and dementia What lies behind the association?. Neurology.

[39] Sharp ES, Gatz M (2011). The relationship between education and dementia an updated systematic review. Alzheimer Dis Assoc Disord.

[40] Fratiglioni L, Paillard-Borg S, Winblad B (2004). An active and socially integrated lifestyle in late life might protect against dementia. Lancet Neurol.

[41] Lourida I, Hannon E, Littlejohns TJ (2019). Association of Lifestyle and Genetic Risk With Incidence of Dementia. JAMA.

[42] Jones DS, Greene JA (2016). Is Dementia in Decline? Historical Trends and Future Trajectories. N Engl J Med.

[43] Volksgezondheidenzorg.info. Dementie C, Context H. 2020. https://www.volksgezondheidenzorg.info/onderwerp/dementie/cijfers-context/huidige-situatie. Accessed April 21st, 2021.

[44] Henri van den Pol RL. Evelien Leegwater. Zorggebruik van mensen met dementie in beeld2019.

