# Supplementary material for: Projected prevalence and incidence of dementia accounting for secular trends and birth cohort effects: a population-based microsimulation study
Source: Eur J Epidemiol. 2022 Jun 22;37(8):807–14. doi: 10.1007/s10654-022-00878-1 (PMC9215138; doi:10.1007/s10654-022-00878-1)
Supplement: Supplementary file 1 — Supplementary Material 1 [file 10654_2022_878_MOESM1_ESM.docx]

**Online Supplement to: C.C. Brück, F.J. Wolters, M.A. Ikram, I.M.C.M. de Kok. Projected prevalence and incidence of dementia accounting for secular trends and birth cohort effects: a population-based microsimulation study**

**European Journal of Epidemiology**

*C.C. Brück^1^, F.J. Wolters^2^, M.A. Ikram^2^, I.M.C.M. de Kok^1^*

^1^Department of Public Health, Erasmus MC University Medical Center, Rotterdam, the Netherlands

^2^Department of Epidemiology, Erasmus MC University Medical Center, Rotterdam, the Netherlands

Correspondence to: Chiara C. Brück, [c.bruck@erasmusmc.nl](mailto:c.bruck@erasmusmc.nl)

**Appendix A: Life tables**

We constructed cohort specific life tables based on historic cohort age- and sex-specific death rates from the years 1910 to 2027 [1, 2], historic period age- and sex-specific death rates from the years 1928 to 2018 [3, 4], projections of age- and sex-specific death rates for the years 2019 to 2070 [5, 6], and predictions for the years 2071 to 2089 based on the historic and projected death rates. Finally, the life tables were adjusted for dementia-related mortality [7]. Figure A1 depicts the resulting female and male life tables for the 10-year birth cohorts 1910-09 to 1980-89.


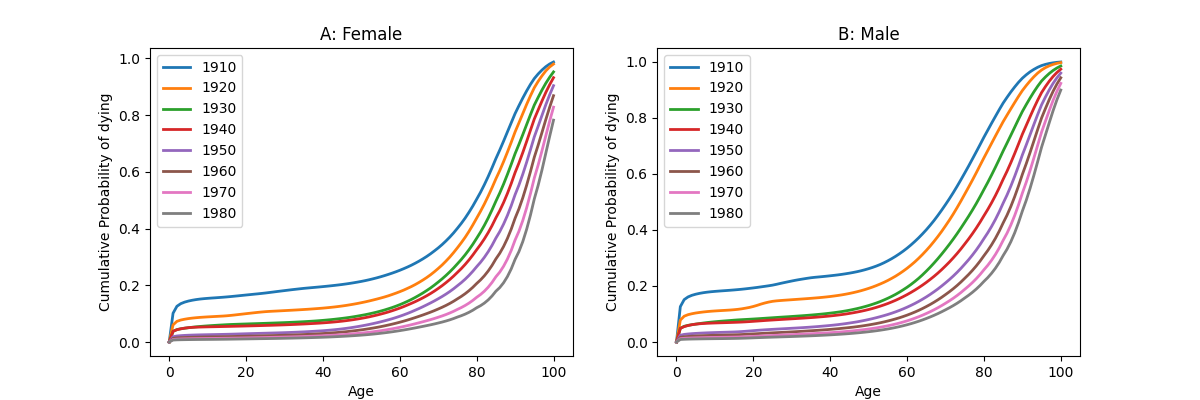


**Figure A1 Construction of cohort specific female (A) and male (B) life tables based on historic data and projections, adjusted for dementia-related mortality.**

Survival of the Dutch birth cohorts has improved over the last century (Figure A1). Similar trends have occurred in other high-income countries such as the United States [8] and the United Kingdom [9]. Figure A2 shows the female and male life tables of the 1910 and 1980 birth cohorts for the Netherlands, the United States, and the United Kingdom. The 1910 life tables are very similar for all three countries. The 1980 life tables show greater differences, with the United States having the highest mortality in older age, then the United Kingdom and finally, the Netherlands having the lowest mortality. Despite these small differences in the 1980 life tables, the general trend of lower mortality in the 1980 birth cohort compared to the 1910 birth cohort is clearly present in all three countries.


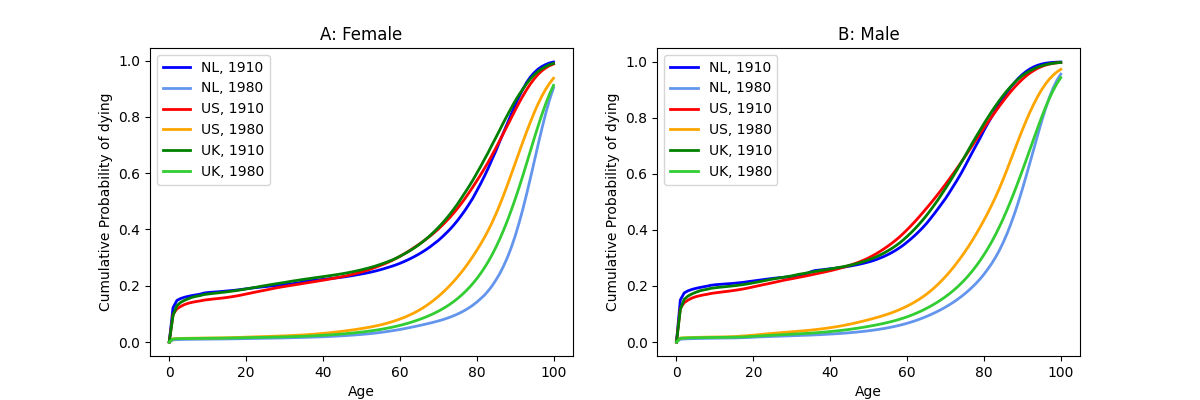


**Figure A2** **Comparison of the 1910 and 1980 birth cohort life tables for the Netherlands (NL), United States (US) and United Kingdom (UK).**

**Appendix B: Model Specifications**

Microsimulation models are mathematical models that provide a powerful tool to account for variability between subjects within a population framework [10, 11], which is necessary for complex diseases such as dementia. Microsimulation models allow individuals to be simulated one at a time (instead of proportions of a cohort), enabling new events to be able to dependent on past events of that individual. Microsimulation models can evaluate uncertainties, take trends in risk factors into account, and estimate long-term and population wide effects of (hypothetical) interventions.

MISCAN-Dementia, coded in Python 3.9, is a stochastic, semi-Markov microsimulation model that predicts how dementia incidence and prevalence will develop until 2050. The model allows for unidirectional stage transitions between cognitively normal, mild cognitive impairment (MCI), dementia, and death due to dementia (Figure B1). In any stage, an individual can also die of other causes. Sex-specific duration estimates for MCI were taken from Vermuntet al. [12] and dementia duration was based on median survival from the Rotterdam Study [13].


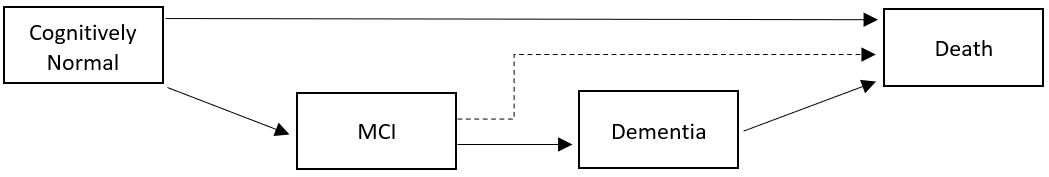


**Figure B1 MISCAN-Dementia model structure.**

MCI = Mild Cognitive Impairment

The distribution of dementia duration by age was modeled with a set of gamma functions. By means of manual calibration the gamma functions were fitted using (1) age specific 1-, 5- and 10-year survival rates from the Rotterdam Study and (2) median survival, which was constructed based on median survival from diagnosis from the Rotterdam Study [13] and a European average for the time duration from onset to diagnosis [14-16]. The following set of gamma distributions was fitted:

Age 50-64: Gamma (shape = 5, scale = 0.25)

Age 65-69: Gamma (shape = 3, scale = 0.4)

Age 70-74: Gamma (shape = 1.75, scale = 0.7)

Age 75-79: Gamma (shape = 1.75, scale = 0.5)

Age 80-84: Gamma (shape = 1, scale = 1.1)

Age 85-100: Gamma (shape = 1, scale = 0.8)

Finally, the model was calibrated to fit the age-specific dementia incidence data of the Rotterdam Study. Parameters of an inverse generalized logistic function and the mean and variance of a hazard function that follows a Gamma distribution were fitted to meet the dementia incidence calibration targets.

**Appendix C: Sensitivity Analysis**

First, we investigated the effect of varying the magnitude of the linear trend by repeating the analysis with the 95% confidence intervals of the linear trend estimate (trends 2.1 and 2.2 in Table C1) [17]. The simulations assuming the lower and upper bounds of the linear trend estimate fit the observed data from the Rotterdam Study well (Figure C1 panel A). The lower bound (7% decline per decade) results in projections closer to the no decline trend, while the upper bound (19% decline per decade) results in even lower projections in incidence than the linear trend of 13% decline per decade.

Second, we relaxed the linearity assumption for the trend in incidence. Three types of nonlinear trends were examined: (1) linear decline across the 1910 and 1940 birth cohorts and decelerating or no decline across the older birth cohorts (trend 3.0 and 3.1 in Table C1); (2) decelerating decline across all birth cohorts (trends 4.0 to 4.4 in Table C1) and (3) accelerating decline across the 1910 and 1940 birth cohorts and decelerating decline across the older birth cohorts (trends 5.0 to 5.2 in Table C1). All nonlinear trend simulations fit the observed data from the Rotterdam Study well (Figure C1 panel B-D). Furthermore, the conclusions presented in the main text are not influenced by varying the nonlinear magnitudes.

| **Trend** | **1910-1920** | **1920-1930** | **1930-1940** | **1940-1950** | **1950-1960** | **1960-1970** | **1970-1980** | **Change (1910-1940)** | **% Change compared to linear** | **Average**  **(1910-1940)** | **% Change compared to linear** |
| --- | --- | --- | --- | --- | --- | --- | --- | --- | --- | --- | --- |
| **No trend (1**.**0)** | 0% | 0% | 0% | 0% | 0% | 0% | 0% | 0% | - | 0.01294 | - |
| **Linear (2.0)** | -13% | -13% | -13% | -13% | -13% | -13% | -13% | 34.1494% | Ref. | 0.01251 | Ref. |
| **Nonlinear (3.0)** | -13% | -13% | -13% | -10% | -7% | -5% | -4% | 34.1497% | 0.00% | 0.01251 | 0.00% |
| **Nonlinear (3.1)** | -13% | -13% | -13% | 0% | 0% | 0% | 0% | 34.1497% | 0.00% | 0.01251 | 0.00% |
| **Nonlinear (4.0)** | -18% | -13% | -8% | -5% | -3% | -1% | 0% | 34.3672% | -0.64% | 0.01276 | -1.97% |
| **Nonlinear (4.1)** | -18% | -13% | -8% | 0% | 0% | 0% | 0% | 34.3672% | -0.64% | 0.01276 | -1.97% |
| **Nonlinear (4.2)** | -22% | -11% | -5% | -4% | -3% | -2% | -1% | 34.0510% | 0.29% | 0.01298 | -3.74% |
| **Nonlinear (4.3)** | -22% | -11% | -5% | 0% | 0% | 0% | 0% | 34.0510% | 0.29% | 0.01298 | -3.74% |
| **Nonlinear (4.4)** | -24% | -12% | -2% | 0% | 0% | 0% | 0% | 34.4576% | -0.90% | 0.01310 | -4.72% |
| **Nonlinear (5.0)** | -8% | -13% | -18% | -13% | -8% | -5% | -3% | 34.3672% | -0.64% | 0.01238 | 1.06% |
| **Nonlinear (5.1)** | -5% | -11% | -22% | -11% | -5% | -3% | -1% | 34.0510% | 0.29% | 0.01245 | 0.52% |
| **Nonlinear (5.2)** | -2% | -12% | -24% | -12% | -2% | 0% | 0% | 34.4576% | -0.90% | 0.01224 | 2.17% |

**Table C1** **Trend assumptions expressed in percentage change between different birth cohorts.**

The simulated age-specific dementia incidence rates irrespective of the birth cohort are compared to the Rotterdam Study data in the data collection period 2002 to 2016 (Figure C1). The simulated dementia incidence rates fit the data very well, with some small deviation for the 90-94 and 95+ groups.


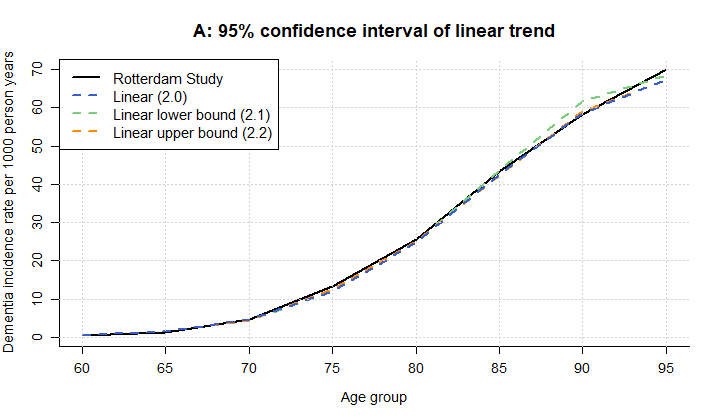

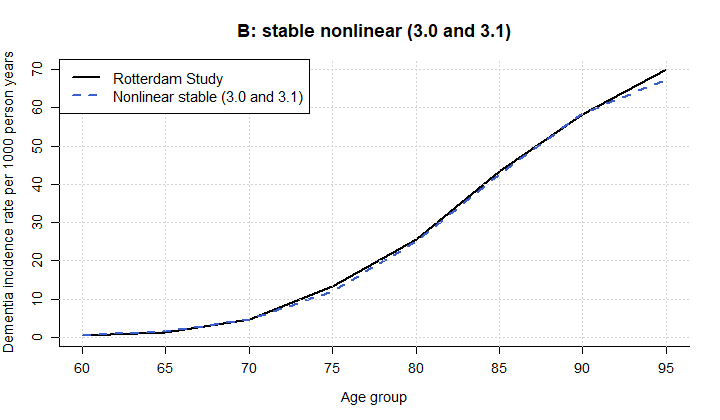

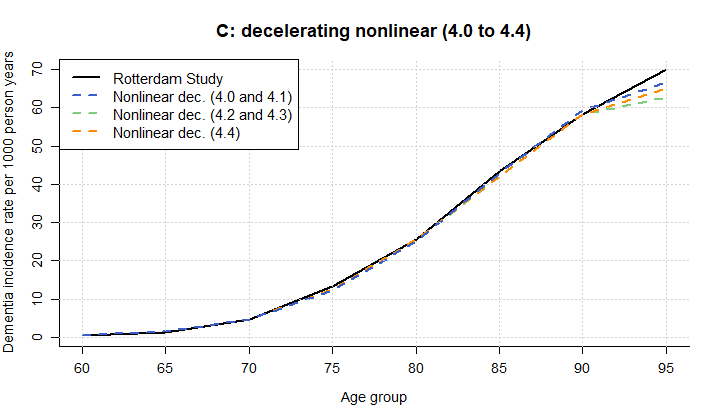

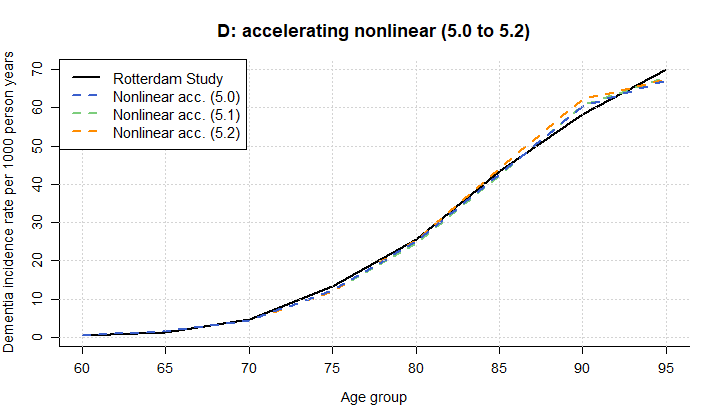


**Figure C1** Age-specific dementia incidence rate per 1000 person years between 2002 and 2016, trend scenario calibration compared to the Rotterdam Study. (A) 95% confidence interval of the linear trend, (B) stable nonlinear (3.0 and 3.1), (C) decelerating nonlinear (4.0 to 4.4), (D) accelerating nonlinear (5.0 to 5.2).


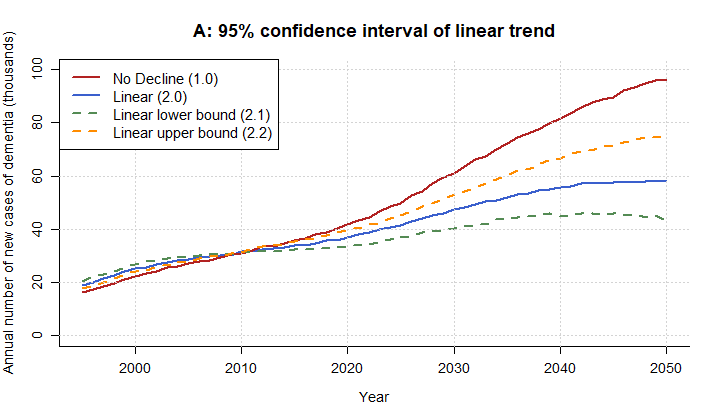

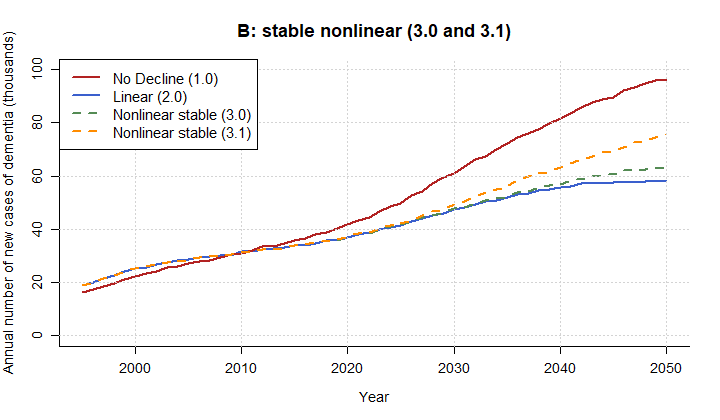

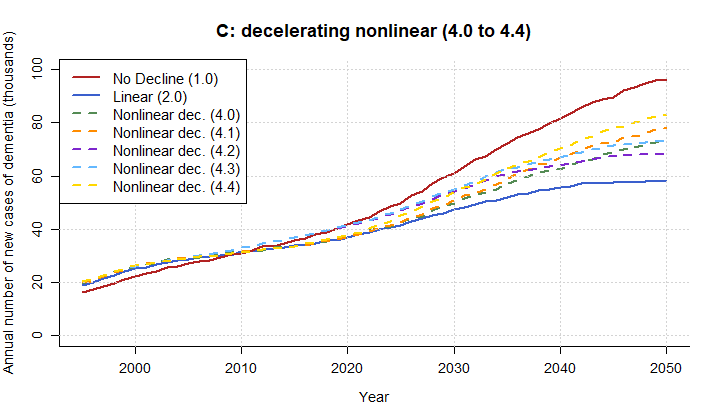

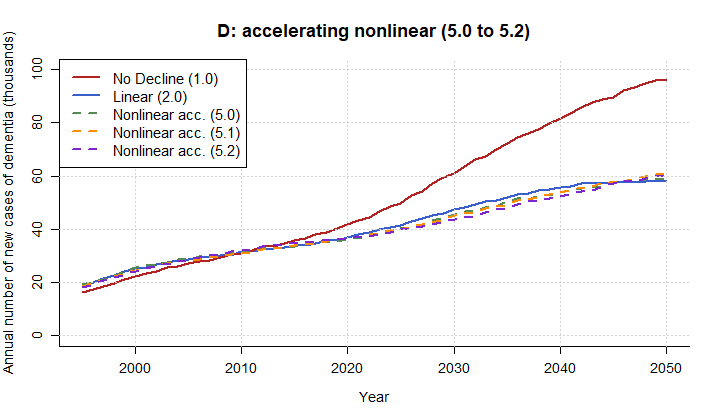


**Figure C2** Total Dutch dementia incidence cases (in thousands) per calendar year, trend scenarios compared to no trend and linear trend. (A) 95% confidence interval of the linear trend, (B) stable nonlinear (3.0 and 3.1), (C) decelerating nonlinear (4.0 to 4.4), (D) accelerating nonlinear (5.0 to 5.2).


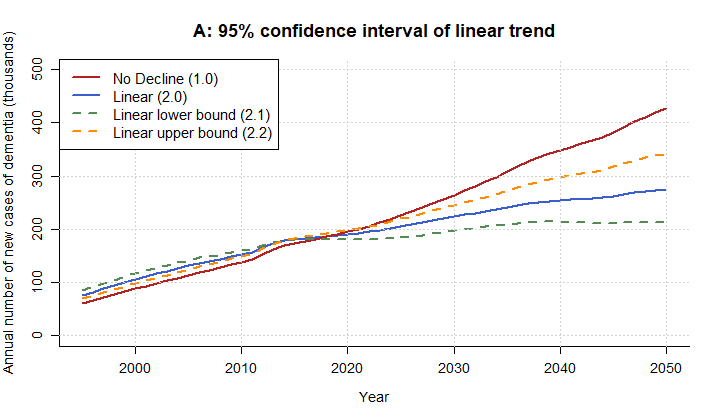

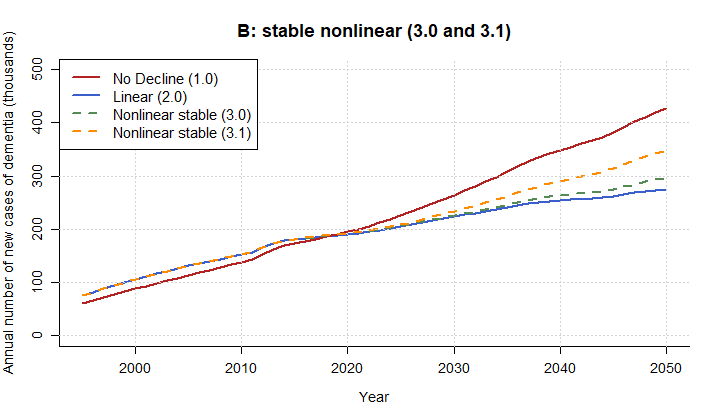

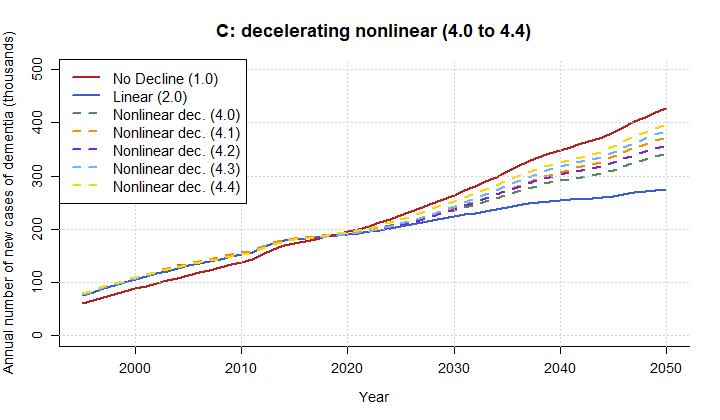

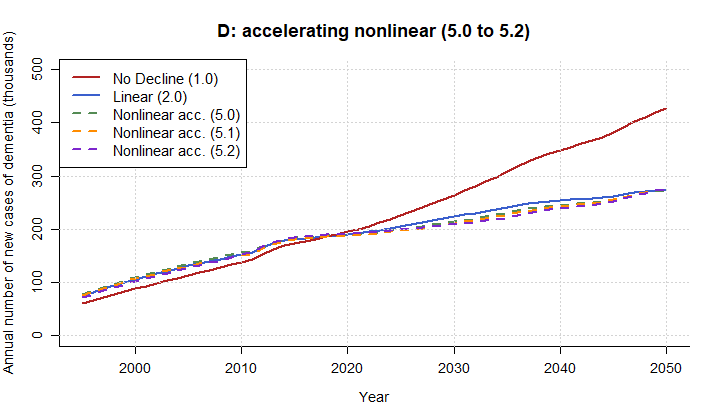


**Figure C3** Dutch dementia prevalence cases (in thousands) per calendar year, trend scenarios compared to no trend and linear trend. (A) 95% confidence interval of the linear trend, (B) stable nonlinear (3.0 and 3.1), (C) decelerating nonlinear (4.0 to 4.4), (D) accelerating nonlinear (5.0 to 5.2).

|  |  | **2030** | | | |  | **2050** | | | |
| --- | --- | --- | --- | --- | --- | --- | --- | --- | --- | --- |
|  | **Trend Scenario** | **Rate** | **Δ no trend** | **Δ linear** | **Increase**  **2020 to 2030** |  | **Rate** | **Δ no trend** | **Δ linear** | **Increase**  **2020 to 2050** |
| **Base Analysis** | No trend (1.0) | 14.4 | *ref* | 28% | **45%** |  | 19.9 | *ref* | 65% | **130%** |
|  | Linear (2.0) | 11.2 | -22% | *ref* | **29%** |  | 12.1 | -39% | *ref* | **58%** |
| **95% CI Linear** | Linear low (2.1) | 12.6 | -13% | 12% | **33%** |  | 15.4 | -23% | 28% | **87%** |
|  | Linear high (2.2) | 9.5 | -34% | -15% | **20%** |  | 9.0 | -55% | -26% | **29%** |
| **Non-linear** | Nonlinear (3.0) | 11.3 | -22% | 0% | **28%** |  | 13.2 | -34% | 9% | **71%** |
|  | Nonlinear (3.1) | 11.6 | -19% | 3% | **32%** |  | 15.6 | -22% | 29% | **102%** |
|  | Nonlinear (4.0) | 11.8 | -18% | 5% | **34%** |  | 15.0 | -25% | 24% | **96%** |
|  | Nonlinear (4.1) | 12.0 | -16% | 7% | **36%** |  | 16.1 | -19% | 34% | **109%** |
|  | Nonlinear (4.2) | 12.8 | -11% | 14% | **31%** |  | 14.2 | -29% | 17% | **66%** |
|  | Nonlinear (4.3) | 13.0 | -9% | 16% | **33%** |  | 15.2 | -24% | 26% | **77%** |
|  | Nonlinear (4.4) | 12.8 | -11% | 14% | **43%** |  | 17.2 | -14% | 42% | **119%** |
|  | Nonlinear (5.0) | 10.8 | -25% | -4% | **26%** |  | 12.3 | -38% | 2% | **65%** |
|  | Nonlinear (5.1) | 10.7 | -25% | -5% | **25%** |  | 12.5 | -37% | 4% | **66%** |
|  | Nonlinear (5.2) | 10.3 | -28% | -8% | **19%** |  | 12.4 | -38% | 3% | **64%** |

**Table C2** Incidence rate (per 1000 life years above 65 years) projections for 2030 and 2050, and relative to the *no decline* and *linear trend* base analysis

|  |  | **2030** | | | |  | **2050** | | | |
| --- | --- | --- | --- | --- | --- | --- | --- | --- | --- | --- |
|  | **Trend Scenario** | **Rate** | **Δ no trend** | **Δ linear** | **Increase**  **2020 to 2030** |  | **Rate** | **Δ no trend** | **Δ linear** | **Increase**  **2020 to 2050** |
| **Base Analysis** | No trend (1.0) | . 62.5 | *ref* | 18% | **35%** |  | . 88.2 | *ref* | 56% | **118%** |
|  | Linear (2.0) | . 52.9 | -15% | *ref* | **17%** |  | . 56.5 | -36% | *ref* | **43%** |
| **95% CI Linear** | Linear low (2.1) | . 58.0 | -7% | 10% | **24%** |  | . 70.8 | -20% | 25% | **72%** |
|  | Linear high (2.2) | . 46.6 | -25% | -12% | **9%** |  | . 43.9 | -50% | -22% | **17%** |
| **Non-linear** | Nonlinear (3.0) | . 53.5 | -14% | 1% | **18%** |  | . 61.4 | -30% | 9% | **55%** |
|  | Nonlinear (3.1) | . 55.3 | -11% | 5% | **22%** |  | . 72.1 | -18% | 28% | **81%** |
|  | Nonlinear (4.0) | . 55.7 | -11% | 5% | **22%** |  | . 70.8 | -20% | 25% | **77%** |
|  | Nonlinear (4.1) | . 56.4 | -10% | 7% | **24%** |  | . 76.9 | -13% | 36% | **93%** |
|  | Nonlinear (4.2) | . 56.2 | -10% | 6% | **25%** |  | . 73.8 | -16% | 31% | **88%** |
|  | Nonlinear (4.3) | . 57.3 | -8% | 8% | **27%** |  | . 79.2 | -10% | 40% | **101%** |
|  | Nonlinear (4.4) | . 59.8 | -4% | 13% | **30%** |  | . 82.0 | -7% | 45% | **103%** |
|  | Nonlinear (5.0) | . 50.7 | -19% | -4% | **13%** |  | . 56.5 | -36% | 0% | **44%** |
|  | Nonlinear (5.1) | . 49.9 | -20% | -6% | **12%** |  | . 57.3 | -35% | 1% | **47%** |
|  | Nonlinear (5.2) | 49.6 | -21% | -6% | **9%** |  | 57.2 | -35% | 1% | **44%** |

**Table C3** Prevalence rate (per 1000 individuals above 65 years) projections for 2030 and 2050, and relative to the *no decline* and *linear trend* base analysis

**References**

1. Human Mortality Database. University of California BU, and Max Planck Institute for Demographic Research (Germany). Netherlands, Life tables (cohort 1x1), Females. 2020. <www.mortality.org>. Accessed March 18th, 2021.

2. Human Mortality Database. University of California BU, and Max Planck Institute for Demographic Research (Germany). Netherlands, Life tables (cohort 1x1), Males. 2020. <www.mortality.org>. Accessed March 18th, 2021.

3. Human Mortality Database. University of California BU, and Max Planck Institute for Demographic Research (Germany). Netherlands, Life tables (period 1x1), Males. 2020. <www.mortality.org>. Accessed March 18th, 2021.

4. Human Mortality Database. University of California BU, and Max Planck Institute for Demographic Research (Germany). Netherlands, Life tables (period 1x1), Females. 2020. <www.mortality.org> Accessed March 18th, 2021.

5. Statistics Netherlands. Prognose periode-levensverwachting; geslacht en leeftijd, 2019-2060. 2020. <https://opendata.cbs.nl/statline/#/CBS/nl/dataset/84647NED/table?ts=1600423898954>. Accessed September 18, 2020.

6. Statistics Netherlands. Prognose periode-levensverwachting; geslacht en leeftijd, 2020-2070. 2020. <https://opendata.cbs.nl/#/CBS/nl/dataset/84883NED/table?searchKeywords=leeftijd>. Accessed March 18th, 2021.

7. Statistics Netherlands. Overledenen; doodsoorzaak (uitgebreide lijst), leeftijd, geslacht. 2020. <https://opendata.cbs.nl/statline/#/CBS/nl/dataset/7233/table?ts=1615054270240>. Accessed April 1st, 2021.

8. Life Tables for the United States Social Security Area 1900-2100 [database on the Internet]2005 [cited June 18th, 2021]. Available from: <https://www.ssa.gov/oact/NOTES/as120/LifeTables_Tbl_7.html>.

9. Past and projected mortality rates (qx) from the 2018-based England and Wales cohort life tables: Principal projection [database on the Internet]2020 [cited June 18th, 2021]. Available from: <https://www.ons.gov.uk/peoplepopulationandcommunity/birthsdeathsandmarriages/lifeexpectancies/adhocs/11272cohortmortalityratesenglandandwales2018basedprincipalprojection>.

10. Kretzschmar M. Disease modeling for public health: added value, challenges, and institutional constraints. J Public Health Policy. 2020;41(1):39-51. <https://doi.org/10.1057/s41271-019-00206-0>

11. Law AM, Kelton WD, Kelton WD. Simulation modeling and analysis: McGraw-Hill New York; 2000.

12. Vermunt L, Sikkes SA, Van Den Hout A, et al. Duration of preclinical, prodromal, and dementia stages of Alzheimer's disease in relation to age, sex, and APOE genotype. Alzheimers Dement. 2019;15(7):888-98. <https://doi.org/10.1016/j.jalz.2019.04.001>

13. Wolters FJ, Tinga LM, Dhana K, et al. Life expectancy with and without dementia: a population-based study of dementia burden and preventive potential. Am J Epidemiol. 2019;188(2):372-81. <https://doi.org/10.1093/aje/kwy234>

14. Bonanni L, Di Giacomo R, D'Amico A, et al. Akinetic crisis in dementia with Lewy bodies. J Neurol Neurosurg Psychiatry. 2016;87(10):1123-6. <https://doi.org/10.1136/jnnp-2015-312914>

15. Oosterveld SM, Kessels RP, Hamel R, et al. The influence of co-morbidity and frailty on the clinical manifestation of patients with Alzheimer's disease. J Alzheimers Dis. 2014;42(2):501-9. <https://doi.org/10.3233/JAD-140138>

16. Stubendorff K, Hansson O, Minthon L, Londos E. Differences in survival between patients with dementia with lewy bodies and patients with Alzheimer's disease - Measured from a fixed cognitive level. Dementia Geriatr Cogn Disord. 2011;32(6):408-16. <https://doi.org/10.1159/000335364>

17. Wolters FJ, Chibnik LB, Waziry R, et al. Twenty-seven-year time trends in dementia incidence in Europe and the United States: The Alzheimer Cohorts Consortium. Neurology. 2020;95(5):e519-e31. <https://doi.org/10.1212/WNL.0000000000010022>
